# Serum Concentrations of Folate Forms Following Supplementation of Multimicronutrients with 400 µg or 800 µg Mix of (6S)‐5‐Methyltetrahydrofolate and Folic Acid (1:1) in Women of Childbearing Age

**DOI:** 10.1002/mnfr.202400444

**Published:** 2024-10-28

**Authors:** Rima Obeid, Elena Rube, Christiane Schön, Jürgen Geisel

**Affiliations:** ^1^ Department of Clinical Chemistry and Laboratory Medicine Saarland University Hospital D‐66420 Homburg/Saar Germany; ^2^ BioTeSys GmbH Schelztor Street 54–56, D‐73728 Esslingen Germany

**Keywords:** folic acid, methylfolate, pregnancy, serum folate, supplements

## Abstract

**Scope:**

A mixture of (6S)‐5‐methyltetrahydrofolate‐calcium salt ((6S)‐5‐MTHF‐Ca) and folic acid (FA) from multimicronutrient supplements may show a dose‐dependent effect on serum folate concentrations.

**Methods and results:**

The study compares fasting concentrations of serum folate spices after 8 weeks of either 400 or 800 µg day^−1^ of 1:1 folate mixture in 172 nonpregnant women. Serum (6S)‐5‐MTHF concentrations raise from a mean (SD) of 19.1 (13.4) to 73.9 (19.6) nmol L^−1^ in the 800 µg group and from 17.5 (9.4) to 54.5 (21.1) nmol L^−1^ in the 400 µg group (*p* < 0.001 within‐group changes). The raise in serum (6S)‐5‐MTHF is stronger in the 800 µg compared to the 400 µg group (*p* < 0.001 between‐group). The prevalence of FA concentrations ≥0.20 nmol L^−1^ increases between baseline and week 8 in both groups, but is not different between the groups (*p* = 0.116). The mean percentage of (6S)‐5‐MTHF of total serum folate increases in both intervention groups, but is not different between the groups at 8 weeks (95.5 (4.1)% versus 94.4 (5.7)%, *p* = 0.309).

**Conclusions:**

Supplementation of multimicronutrients with 800 µg folate mix for 8 weeks causes higher serum (6S)‐5‐MTHF concentrations, but not a higher prevalence of detectable FA compares to 400 µg folate.

## Graphical abstract

People receive 800 or 400 µg of 1:1 mixture of (6S)‐5‐methyltetrahydrofolate‐calcium ((6S)‐5‐MTHF‐Ca) and folic acid (FA) from multimicronutrient supplements for 8 weeks. The high dose causes a stronger increase in serum (6S)‐5‐MTHF than the low dose. The 400 µg day^−1^ FA does not cause a higher prevalence of detectable serum unmetabolized FA than 200 µg FA.

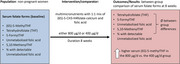

## 1 Introduction

Using folic acid (FA) from fortified foods or food supplements increases maternal blood folate and may reduce the risk of neural tube defects (NTDs) in the offspring.^[^ ^1^, ^2^ ^]^ Women planning pregnancy and pregnant women are recommended to take supplemental folate on top of the usual diet until the end of the first trimester.^[^ ^3^ ^]^ In women of fertile age, the concentrations of red blood cell folate (RBC‐folate) and serum folate should optimally be above 906 nmol L^−1[^ ^4^ ^]^ and 25 nmol L^−1^,^[^ ^5^ ^]^ respectively. Achieving optimal serum folate concentrations may be more difficult in women with vitamin B12 deficiency.^[^ ^5^ ^]^ Most antenatal supplements provide between 400 and 1000 µg folate and other nutrients such as vitamins B12 and B6.

Using folate supplements may lead to exceeding the Upper Tolerable Level (UL) (1 mg folate per day) in a considerable number of women, especially those from countries with mandatory fortification with FA.^[^ ^6^, ^7^ ^]^ FA is not present in blood unless taken through fortified foods or food supplements.^[^ ^8^ ^]^ Exposure to >200 µg FA can lead to the appearance of unmetabolized FA (UMFA) in serum/plasma,^[^ ^8^, ^9^, ^10^, ^11^ ^]^ cord plasma of the newborns,^[^ ^10^, ^11^, ^12^, ^13^, ^14^, ^15^ ^]^ and in human milk.^[^ ^16^ ^]^ High‐dose supplemental FA during pregnancy has been related to some undesirable health outcomes.^[^ ^17^, ^18^, ^19^, ^20^, ^21^ ^]^ For example, a study in women with epilepsy has shown that taking ≥1 mg day^−1^ FA during pregnancy is associated with an increased risk of childhood cancer.^[^ ^19^ ^]^ High‐dose FA in pregnant animals may negatively affect fetal development^[^ ^20^ ^]^ or cause pseudo‐deficiency of the methylenetetrahydrofolate reductase (MTHFR) enzyme.^[^ ^21^ ^]^ The presence of UMFA (a nonphysiological form) in the circulation could explain some undesirable effects of high‐dose FA.

Natural foods contain predominantly (6S)‐5‐methyltetrahydrofolate ((6S)‐5‐MTHF). In addition, (6S)‐5‐MTHF constitutes approximately 88%–95% of total folate in serum and red blood cells.^[^ ^8^ ^]^ In contrast to FA that needs to be enzymatically reduced to THF, (6S)‐5‐MTHF is readily available to support folate metabolism or for storage. In repeated‐dose human studies, supplementation of (6S)‐5‐MTHF‐calcium salt ((6S)‐5‐MTHF‐Ca) has been shown to increase circulating concentrations of folate at least to the same extent as when an equimolar dose of FA is supplemented.^[^ ^22^, ^23^ ^]^ At doses ≥400 µg day^−1^, (6S)‐5‐MTHF‐Ca has higher bioavailability compared to FA (reviewed in ref. [24]). Approximately 25%–30% of antenatal products on the US market contain a (6S)‐5‐MTHF salt as a sole source of folate and 25% contain a mixture of (6S)‐5‐MTHF salt and FA,^[^ ^25^ ^]^ suggesting implications of the supplemental folate form on achieving optimal circulating folate.

Like folate, vitamin B12 and vitamin B6 contribute to normal one‐carbon metabolism, including homocysteine (Hcy) metabolism.^[^ ^26^, ^27^ ^]^ Most observational and interventional studies on the association between FA intake and serum UMFA did not investigate vitamin B12 and B6 intakes or statuses.^[^ ^13^ ^]^ In a randomized controlled trial (RCT) in elderly people, a daily dose of 400 µg FA over 3 weeks was more likely to cause detectable concentrations of UMFA in blood compared to the same FA dose in combination with 10 µg vitamin B12 and 8 mg vitamin B6.^[^ ^28^ ^]^ These results suggest that FA should be combined with vitamin B12 and B6 to avoid UMFA in serum.

Serum concentrations of folate forms such as (6S)‐5‐MTHF and UMFA have not been investigated after supplementing (6S)‐5‐MTHF‐Ca along with FA and other nutrients. We hypothesized that repeated intake of antenatal multimicronutrients providing 400 or 800 µg day^−1^ of a mixture of 1:1 (6S)‐5‐MTHF‐Ca and FA (thus providing 200 or 400 µg day^−1^ FA) may cause a dose‐dependent increase in serum concentrations of folate forms. We conducted a secondary analysis of data from a previous RCT in nonpregnant women^[^ ^29^ ^]^ to compare serum concentrations of folate forms between women who received multimicronutrients containing either 400 or 800 µg day^−1^ folate mixture for 8 weeks.

## 2 Experimental Section

### 2.1 Subjects

The randomized open‐labeled controlled study was conducted between January and May 2016 at BioTeSys GmbH, Esslingen am Neckar, Germany. The inclusion criteria were: women sex, age ≥18 and ≤45 years, body mass index between 17 and 30 kg m^−^^2^, and good physical and mental health. Pregnant and lactating women and those planning to become pregnant during the study were not eligible to participate. Changes of serum and RBC‐folate concentrations and plasma Hcy constituted the secondary outcomes of the study.^[^ ^30^, ^31^ ^]^ The study is registered at the German Clinical Trial Register; DRKS‐ID: DRKS00009770.

The protocols of the original and the present studies were reviewed and approved by the Medical Ethics Commission of the Baden‐Württemberg region, Germany (approval numbers: F‐2015‐102 and F‐2021‐172). The study was conducted according to the ethical principles for medical research involving human subjects as stated in Helsinki Declaration. All participants were informed about the study purpose, and they provided their signed consent to the study.

### 2.2 Dosage Information/Dosage Regimen

Women (*n* = 201) were randomized to receive a daily oral multimicronutrient supplement containing either 400 µg (*n* = 100) or 800 µg (*n* = 101) mixture of (6S)‐5‐MTHF‐Ca and FA (ratio 1:1). The participants took a daily capsule containing 400 µg folate ((6S)‐5‐CH3‐H4folate‐Ca and FA [1:1]) (Elevit® gynvital) or a tablet containing 800 µg folate ((6S)‐5‐CH3‐H4folate‐Ca and FA [1:1]) (Femibion® 1). The study products differed in several other nutrients (Suppporting Information Table S1). For example, compared to Elevit® gynvital, Femibion® 1 contained 0.9 µg more B12, 0.2 mg more B2, 30 µg more biotin, but did not contain vitamin A, zinc, omega 3‐fatty acids, or iron.

The participants were randomized to receive one of the study products. All women started the intervention at baseline visit and five women (two in the 400 µg group and three in the 800 µg group) did not return for the final study visit after 8 weeks, thus 98 women in each study group had completed the intervention. Compliance with the study supplementation was documented by counting the tablets or capsules at each study visit (after 4 and 8 weeks of starting the intervention) and additionally confirmed by checking the study diary where participants had to document their daily intake of the supplements. The compliance rate was 99.3% and 99.6% in the 400 and 800 µg, respectively. None of the participants had less than 80% compliance that was the criteria for considering the participant incompliant.

The folate intake from the supplements was calculated as µg dietary folate equivalent (DFE) according to the recent recommendation of the European Food Safety Authority (EFSA) by using the same conversion factor of 1.7 for both FA and (6S)‐5‐MTHF‐Ca when the intake is less than 400 µg.^[^ ^24^ ^]^ When the intake is 400 µg, the conversion factors are 1.7 for FA and 2.0 for (6S)‐5‐MTHF‐Ca to account for a higher bioavailability of (6S)‐5‐MTHF‐Ca compared to FA at this intake level.^[^ ^24^ ^]^ Thus, the study products contained either 680 µg (200 * 1.7 + 200 * 1.7) or 1480 µg (400 * 1.7 + 400 * 2.0) of DFEs. The folate content in the supplements would be 680 and 1360 µg DFE according to the US Food and Drug Administration (FDA) that assumes the same conversion factor for FA and (6S)‐5‐MTHF‐Ca to DFE.

### 2.3 Samples and Biomarkers

Fasting (≥10 h) blood samples were collected at baseline visit, week 4 and week 8. In the present study, samples from the baseline visit and the end visit (week 8) were used. Participants were asked to abstain from the study products 24 h before the blood collection. Blood samples were collected into dry tubes and tubes prefilled with K + EDTA. Blood samples were centrifuged and the serum or EDTA‐plasma were separated after 30 min of blood collection. Blood samples were immediately frozen at –80 °C until analyses of the biomarkers. Stored novel serum samples from the baseline and 8 week visits were used for measurement of folate forms. All samples from the same participant were measured in the same run to minimize the effect of day‐to‐day analytical variations.

**Figure 1:**
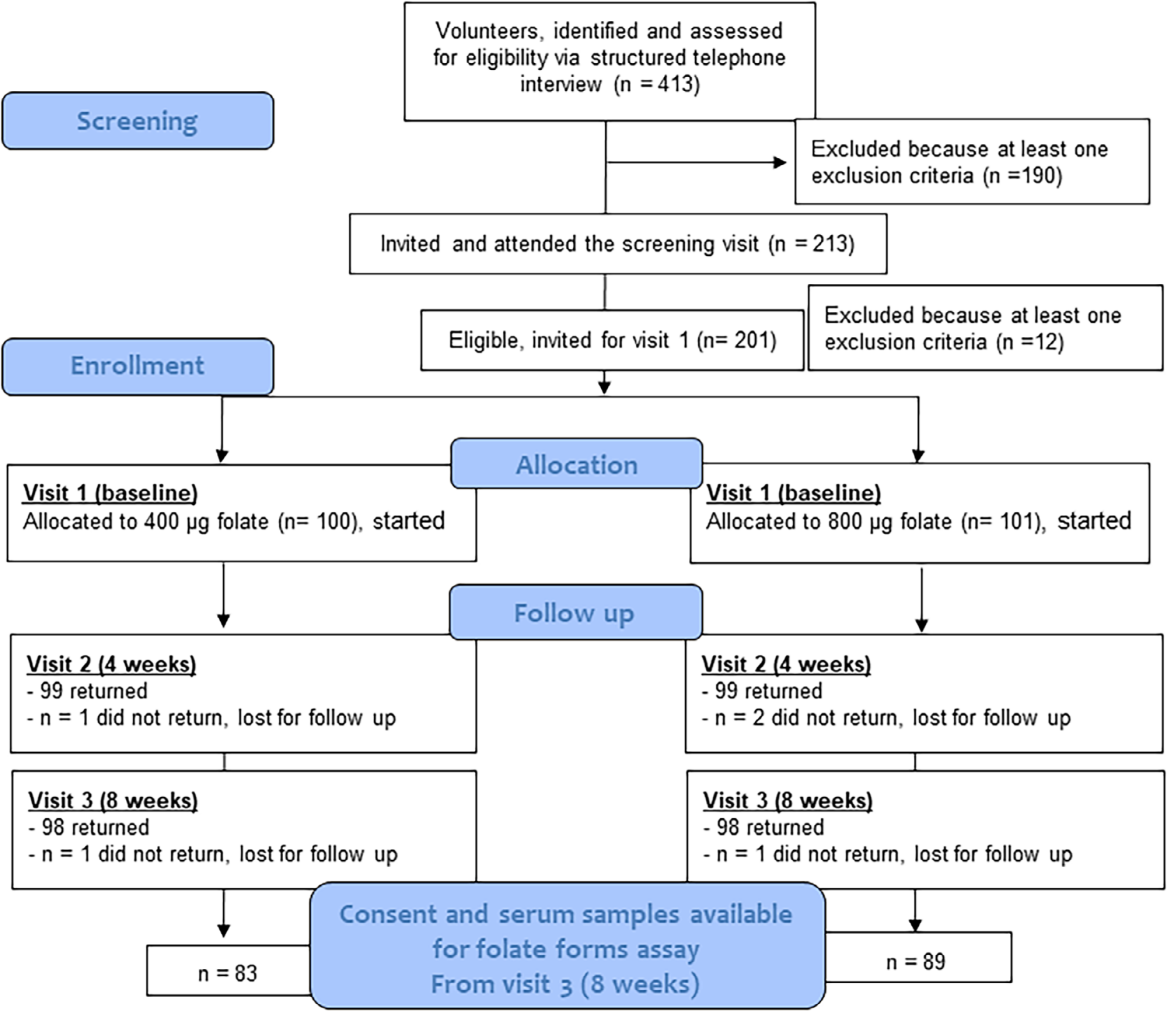
Study flow diagram.

The present study included 172 women who provided their consent for the additional blood analyses and where blood samples were available (**Figure** **1**, Study flow diagram). Concentrations of plasma Hcy (measured using HPLC method) and those of total serum folate and whole blood folate (measured using reagents from Immulite®) have been reported elsewhere.^[^ ^31^ ^]^

Serum concentrations of (6S)‐5‐MTHF, tetrahydrofolate (THF), 5‐formyltetrahydrofolate (5‐formylTHF), UMFA, and 5,10‐methenyltetrahydrofolate (5,10‐MethenylTHF) were measured using an Acquity Ultra Performance LC system coupled to a MicroMass Quattro Premier XE tandem quadrupole mass spectrometer (UPLCMS‐MS, Waters Corporation, Milford, MA, USA) at the Central laboratory of the University Hospital of the Saarland, Homburg, Germany as reported before.^[^ ^32^ ^]^ The sum of the measured concentrations of folate forms using the UPLCMS‐MS assay was computed and considered as total serum folate. In addition, the percentage of each of the folate forms of total serum folate was computed. When the concentrations of the minor folate forms were below the limit of detection (LOD), they were not set to zero, but values were used in the data analysis as measured. The LOD for UMFA is 0.20 nmol L^−1^ (signal‐to‐noise ratio ≥5), and the limit of quantification (LOQ, signal‐to‐noise ratio ≥10) is 0.40 nmol L^−1^. The between‐day coefficients of variation (CV%) for the UPLCMS‐MS method were <10% for (6S)‐5‐MTHF concentrations and <15% for the other folate forms.

### 2.4 Statistical Analyses

The statistical analyses were conducted using the IBM® SPSS® Statistics package Version 32 (SPSS, Inc., Chicago, IL, USA). The descriptive results are shown as mean (standard deviation [SD]) for continuous variables and as absolute numbers (*n*) and relative frequencies (%) for categorical variables.

The distribution of the continuous variables was studied using the one‐sample Kolmogorov–Smirnov test and Lilliefors significance correction. Q–Q plots were additionally used to evaluate the distribution of the data. Concentrations of all folate forms were not normally distributed.

The Mann–Whitney test was used to study the differences in continuous variables between the intervention groups at baseline or 8 weeks. In addition, the change of (6S)‐5‐MTHF between baseline and week 8 (concentrations at week 8 minus those at baseline) were computed for each participant. The changes of (6S)‐5‐MTHF were compared between the study groups using an ANCOVA test that was adjusted for individual baseline concentrations of (6S)‐5‐MTHF. The differences in the distribution of binary variables between the study groups were investigated by using the Chi‐square test.

The paired *t*‐test was applied on the log_10_‐transformed values of (6S)‐5‐MTHF concentrations to study the within‐group changes (pre–post comparisons). Differences in the minor folate forms within each group (pre–post comparisons) were studied using the nonparametric Wilcoxon signed‐rank test. The McNemar test was used to study the within‐group differences in the percentage of women with detectable UMFA between baseline and week 8.

Due to the very low concentrations of the minor folate forms (either zero or below the quantification limit in the majority of samples), we did not compute the within‐individual differences or applied statistical tests on such differences. *p* values ≤ 0.05 were considered to indicate statistical significance.

## 3 Results

The study included 172 nonpregnant women with a mean (SD) age of 26.9 (6.5) years. Additional characteristics are shown in Supporting Information Table S2.

The concentrations of (6S)‐5‐MTHF at baseline were not different between the groups (17.5 (9.4) nmol L^−1^ in the 400 µg group and 19.1 (13.4) nmol L^−1^ in the 800 µg group; between‐group *p* = 0.386). At 8 weeks, the serum concentrations of (6S)‐5‐MTHF were 54.5 (21.1) nmol L^−1^ in the women who received 400 µg day^−1^ folate and 73.9 (19.6) nmol L^−1^ in those who received 800 µg day^−1^ folate (between‐group *p* < 0.001) (**Table** **1**). The mean (SD) of individual changes of (6S)‐5‐MTHF concentrations from baseline to week 8 were 37.0 (17.2) nmol L^−1^ and 54.8 (17.4) nmol L^−1^ in the 400 and the 800 µg group, respectively (the between‐group *p* value < 0.001, ANCOVA test adjusted for baseline (6S)‐5‐MTHF concentrations).

**Table 1:** Concentrations of serum folate species and the proportion of the individual forms of total serum folate at baseline and after 8 weeks of supplementing either 400 or 800 µg day^−1^ of a 1:1 mix of (6S)‐5‐CH3‐H4folate‐Ca and folic acid.

	1:1 Mix of (6S)‐5‐CH3‐H4folate‐Ca and folic acid							
	400 µg day^−1^ (total daily folate intake 680 µg DFE)^a)^, *n* = 83			800 µg day^−1^ (total daily folate intake 1480 µg DFE)^a)^, *n* = 89				
	Baseline	8 weeks	*p* (within‐group)^b)^	Baseline	8 weeks	*p* (within‐group)^b)^	*p* between‐groups at baseline^c)^	*p* between‐groups at 8 weeks^c)^
(6S)‐5‐MTHF [nmol L^−1^]	17.5 (9.4)	54.5 (21.1)	<0.001	19.1 (13.4)	73.9 (19.6)	<0.001	0.386	<0.001
THF [nmol L^−1^]	1.30 (1.83)	2.10 (3.30)	0.018	0.99 (0.97)	2.60 (2.60)	<0.001	0.608	0.004
5‐FormylTHF [nmol L^−1^]	0.07 (0.15)	0.08 (0.18)	0.506	0.11 (0.21)	0.08 (0.17)	0.097	0.276	0.883
UMFA [nmol L^−1^]	0.09 (0.21)	0.14 (0.26)	<0.001	0.10 (0.24)	0.20 (0.29)	<0.001	0.824	0.087
UMFA ≥ 0.20 nmol L^−1^ ^d)^, *n*/total [%]	7/83 [8.4%]	11/83 [13.3%]	0.125^e)^	11/89 [12.4%]	21/89 [23.6%]	0.002^e)^	0.461^f)^	0.116^f)^
UMFA ≥ 0.40 nmol L^−1^ ^g)^, n/total [%]	–	4/83 [4.8%]	–	–	5/89 [5.6%]	–	–	0.814^f)^
5,10‐MethenylTHF [nmol L^−1^]	0.69 (2.00)	0.88 (2.47)	0.394	0.36 (0.94)	0.79 (3.27)	0.446	0.509	0.599
Non‐methylTHF [nmol L^−1^]	2.11 (3.34)	3.2 (3.90)	0.028	1.56 (1.30)	3.70 (4.30)	<0.001	0.519	0.143
Total serum folate (UPLCMS‐MS)^h)^ [nmol L^−1^]	19.6 (10.0)	57.7 (21.6)	< 0.001	20.6 (13.7)	77.6 (21.1)	<0.001	0.792	<0.001
Proportion of folate forms of total serum folate								
(6S)‐5‐MTHF [%]	89.1 (10.9)	94.4 (5.7)	<0.001	91.4 (7.0)	95.5 (4.1)	<0.001	0.405	0.309
THF [%]	7.00 (7.20)	3.71 (4.77)	<0.001	5.70 (5.53)	3.30 (2.9	<0.001	0.358	0.636
5‐FormylTHF [%]	0.34 (0.78)	0.12 (0.31)	0.002	0.61 (1.26)	0.10 (0.21)	<0.001	0.248	0.991
UMFA [%]	0.38 (1.00)	0.23 (0.43)	0.048	0.55 (1.42)	0.26 (0.38)	0.023	0.767	0.219
5,10‐MethenylTHF [%]	3.25 (8.33)	1.53 (4.00)	0.102	1.76 (4.50)	0.85 (2.92)	0.112	0.469	0.359
Non‐methylTHF [%]	10.9 (10.9)	5.6 (5.7)	<0.001	8.6 (7.0)	4.5 (4.1)	<0.001	0.405	0.309

Data are mean (SD) if not otherwise specified. DFE, dietary folate equivalent; FA, folic acid; LO(6S)‐5‐MTHF, (6S)‐5‐methyltetrahydrofolate; SD, standard deviation; THF, tetrahydrofolate; UMFA, unmetabolized folic acid; UPLCMS‐MS, ultra pressure liquid‐chromatography tandem mass spectrometry.

^a)^ The supplements provided (200 µg FA * 1.7 + 200 µg (6S)‐5‐CH3‐H4folate‐Ca * 1.7) = 680 µg DFE or (400 µg FA* 1.7 + 400 µg (6S)‐5‐CH3‐H4folate‐Ca * 2.0) = 1480 µg DFE of folate per day according to the European Food Safety Authority (EFSA)^[^ ^24^ ^]^;

^b)^ *p* values for within‐group comparisons of the means (pre–post comparison) are according to paired *t*‐test applied on the log‐transformed (6S)‐5‐MTHF concentrations. The Wilcoxon signed‐rank test is used to study within‐group differences in the minor forms (UMFA, THF, 5‐formylTHF, and 5,10‐MethenylTHF);

^c)^ Mann–Whitney test is used to compare the continuous variables between the independent intervention groups;

^d)^ The assay limit of detection (signal‐to‐noise ratio ≥ 5) for UMFA is 0.20 nmol L^−1^, the limit of quantification (signal‐to‐noise ratio ≥ 10) is 0.40 nmol L^−1^;

^e)^ McNemar test is used to study the within‐group differences in the proportion of participants with UMFA ≥ 0.20 nmol L^−1^;

^f)^ Chi‐square test is used to compare the proportions of participants with detectable UMFA between the intervention groups;

^g)^ None of the participants had UMFA ≥ 0.40 nmol L^−1^ at baseline;

^h)^ Total serum folate UPLCMS‐MS is the sum of all five folate forms ((6S)‐5‐MTHF, THF, 5‐formylTHF, UMFA, and 5,10‐MethenylTHF).

Moreover, the baseline concentrations of THF, 5‐formylTHF, UMFA, 5,10‐MethenylTHF, and the sum of these non‐methyTHF forms did not differ between the study groups (Table 1). At 8 weeks, the concentrations of the minor folate forms (except for THF) and the non‐methylTHF did not differ between the intervention groups (Table 1).

At baseline, the serum concentrations of UMFA were ≥0.20 nmol L^−1^ (LOD) in 7 of the 83 women (8.4%) in the 400 µg group and in 11 of the 89 women (12.4%) in the 800 µg group (between‐group *p* = 0.461) (Table 1). None of the women had concentrations of UMFA >0.40 nmol L^−1^ (the LOQ of UMFA) at baseline. After 8 weeks of the intervention, 13.3% and 23.6% of the women in the 400 and the 800 µg groups, respectively, had UMFA ≥ 0.20 nmol L^−1^ (between‐group *p* = 0.116) (Table 1). The proportion of women who had serum UMFA ≥ 0.20 nmol L^−1^ only at the end of the intervention, but not at baseline was 4.8% (4 of 83) and 11.2% (10 of 89) in the 400 and 800 µg group, respectively (between‐group *p* = 0.165). Four women in the 400 µg group (4.8%) and 5 in the 800 µg group (5.6%) had UMFA >0.40 nmol L^−1^ at the end of the intervention (between‐group *p* = 0.814) (Table 1).

The percentage of (6S)‐5‐MTHF of total folate did not differ between the groups at baseline (mean [SD] = 89.1 (10.9)% and 91.4 (7.0)% in the 400 and the 800 group, respectively [between‐group *p* = 0.405]) (Table 1). The percentage of (6S)‐5‐MTHF of total folate increased within each of the groups from baseline to 8 weeks (<0.001 within each group) and remained not different between the groups at 8 weeks (*p* = 0.309). The percentages of the non‐methylTHF to total folate declined significantly within each of the study groups from baseline to 8 weeks, but they did not differ between the groups at 8 weeks (between‐group *p* = 0.309).

In summary, the increase in serum (6S)‐5‐MTHF concentrations after 8 weeks was stronger in the high dose than in the low dose folate group. Whereas, the percentage of (6S)‐5‐MTHF to total folate did not differ between the groups at 8 weeks. In addition, the prevalence of detectable UMFA did not differ between the groups at 8 weeks.

There were no significant correlations between the concentrations of UMFA at 8 weeks or the percentage of UMFA of total serum folate and the concentrations of RBC‐folate at 8 weeks or the change of RBC‐folate concentrations from the baseline visit (data not shown).

## 4 Discussion

This study has shown that the daily use of supplements containing 400 or 800 µg folate as a 1:1 mixture of (6S)‐5‐MTHF‐Ca and FA in combination with other B‐vitamins caused a dose‐dependent increase in serum concentrations of (6S)‐5‐MTHF. (6S)‐5‐MTHF was the predominant folate form in serum at baseline and after 8 weeks of using either folate mixture. In addition, the daily intake of 800 µg folate from multimicronutrient supplements (providing 400 µg day^−1^ FA) did not cause a higher prevalence of detectable UMFA in serum compared to the daily intake of 400 µg of folate supplements (providing 200 µg day^−1^ FA). The raise in serum concentrations of UMFA was not proportional to the raise in concentrations of (6S)‐5‐MTHF or to RBC‐folate concentrations (as a marker of folate storage). The results suggest that multimicronutrient supplements containing a mix of (6S)‐5‐MTHF‐Ca, FA, vitamin B6, and vitamin B12 could be used for women planning pregnancy and pregnant women to avoid exaggerated raise of UMFA in serum and to simultaneously ensure sufficient increase of serum (6S)‐5‐MTHF. Such multimicronutrient supplements could be more relevant in situations where folate intake ≥400 µg day^−1^ may be advantageous (e.g., in women with previous adverse pregnancy outcomes) and in women who are exposed to additional FA intake from mandatory food fortification programs.

Early human intervention trials that aimed at reducing the risk of NTDs have used FA,^[^ ^2^, ^33^ ^]^ but not the new generation of active folate such as (6S)‐5‐MTHF salts. The reduction of the risk of NTDs likely functions through raising maternal circulating folate concentrations. Some clinical studies have used (6S)‐5‐MTHF salts as a sole source of folate in antenatal supplements^[^ ^34^ ^]^ or at late pregnancy until lactation.^[^ ^16^ ^]^ A daily intake of (6S)‐5‐MTHF‐Ca for 4–24 weeks in healthy adults from different populations has been shown to be effective in raising folate status markers.^[^ ^23^, ^35^, ^36^, ^37^ ^]^ (6S)‐5‐MTHF‐Ca is at least equal to FA in raising women folate status,^[^ ^38^ ^]^ and it can additionally avoid the appearance of UMFA in the circulation. (6S)‐5‐MTHF‐Ca is directly used in one‐carbon metabolism and in contrast to FA, it does not require oxidation by dihydrofolate reductase (DHFR). DHFR has a limited capacity to reduce FA into active folate, suggesting that high‐dose FA may not be completely converted to active folate.^[^ ^39^ ^]^

In a double blind RCT, Cochrane et al. randomized Canadian pregnant women from weeks 8–21 of gestation to 600 µg day^−1^ FA or an equimolar dose (625 µg) of (6S)‐5‐MTHF‐Ca for a duration of 16 weeks.^[^ ^40^ ^]^ In addition, all participants received multivitamin supplements without FA. In this study, the mean total folate intake (from foods and supplements) was 1200 µg DFE per day. The serum concentrations of (6S)‐5‐MTHF were high in both groups and did not differ significantly between women who received FA and those who received (6S)‐5‐MTHF‐Ca (median 70 versus 78 nmol L^−1^) after 16 weeks of supplementation.^[^ ^40^ ^]^ In contrast, serum UMFA was higher in the FA group (providing 600 µg day^−1^ in addition to a small amount of FA from fortified foods) compared to the (6S)‐5‐MTHF group (adjusted mean difference [95% confidence intervals] = 0.6 [0.2, 1.1 nmol L^−1^]).^[^ ^40^ ^]^ In our study, nonpregnant women who received 800 µg day^−1^ folate (of which 400 µg day^−1^ as FA) for 8 weeks had similar serum concentrations of (6S)‐5‐MTHF, but lower concentrations of UMFA compared to the Canadian women who received 600 µg day^−1^ FA for 16 weeks.^[^ ^40^ ^]^ However, the present study and the Canadian one differ in several aspects. First, in contrast to our participants, the Canadian women were pregnant (started the intervention at gestational age 8–21 weeks).^[^ ^40^ ^]^ Pregnancy is associated with accelerated folate catabolism and excretion.^[^ ^41^ ^]^ Second, the Canadian study used a higher dose of FA and the intervention lasted twice longer than in the present study, suggesting that a higher dose of FA or a longer supplementation period may show different results. Third, genetic variants in folate metabolizing genes may differ across populations and may influence the response to folate supplement.^[^ ^42^ ^]^ Finally, preanalytical conditions such as the time interval between the last supplement and the blood collection (24 h in our study), or fasting hours can contribute to the between‐study variations.

In contrast to the US and Canada,^[^ ^43^, ^44^ ^]^ no mandatory fortification with FA is applied in Germany. This might explain a generally lower prevalence of detectable serum concentrations of UMFA in the present study (10.5% of all fasting blood samples at baseline). In a study on UK pregnant women (prior to starting the mandatory fortification with FA in 2021), 31%–36% of the participants had detectable UMFA in plasma (LOD of the assay = 0.27 nmol L^−1^).^[^ ^10^ ^]^ The presence of UMFA in serum in our nonpregnant participants or in the UK pregnant women could be due to voluntary fortification of some food products with FA or to pregnancy‐related physiological changes in folate homeostasis.

The lack of a dose‐dependent effect of repeated FA intake (200 versus 400 µg day^−1^ from the 400 and 800 µg folate mixtures, respectively) on serum concentrations of UMFA in this study may be due to the simultaneous intake of vitamin B6 and B12 that enhance folate metabolism. A higher intake of vitamin B12 (+0.9 µg) in the high‐dose group may also contribute to the metabolism of the additional amount of FA. In a previous RCT, we supplemented either 400 µg day^−1^ FA alone or in combination with the other B‐vitamins to a group of elderly people for 23 days.^[^ ^28^ ^]^ Thirty‐three percent of the elderly people had detectable concentrations of UMFA in serum after the intervention^[^ ^28^ ^]^ (versus 23.6% in young women from the present study), which could be explained by higher age^[^ ^45^ ^]^ and renal insufficiency^[^ ^46^ ^]^ that are generally associated with higher serum concentrations of UMFA. Sweeney et al. suggested that UMFA is likely to increase in serum when FA intake exceeds a certain threshold.^[^ ^9^ ^]^ Intake of bread providing 400 µg day^−1^ FA for 14 weeks led to the appearance of UMFA in serum which was not the case for lower FA intakes (200 and 100 µg day^−1^).^[^ ^9^ ^]^ Finding no difference in the percentage of women with detectable UMFA after 8 weeks of supplementation of 200 or 400 µg FA in our study suggests that it is theoretically possible that the threshold of FA intake to cause detectable UMFA in serum may be higher in people with adequate B12 and B6 intakes than in those with low intakes of these nutrients.

## 5 Conclusions

Multimicronutrient supplements providing 800 µg day^−1^ of 1:1 mixture of (6S)‐5‐MTHF‐Ca and FA for 8 weeks caused higher increase in serum concentrations of (6S)‐5‐MTHF compared to the 400 µg day^−1^ group. The intervention caused an increase in the percentage of serum (6S)‐5‐MTHF to total folate and a decline in the percentage of non‐methylTHF to total folate. Supplementation of 200 or 400 µg day^−1^ FA from the 400 or the 800 µg mixture of FA and (6S)‐5‐MTHF‐Ca, respectively caused an increase in the prevalence of detectable UMFA in both groups after 8 weeks compared to baseline, but the prevalence of detectable UMFA did not differ between the groups at 8 weeks. Future studies may investigate whether similar results can be obtained in pregnant women and whether supplementing the folate mix with vitamin B6 and B12 may reduce detectable UMFA in populations with additional exposure to FA from fortified foods.

## Conflict of Interest

R.O. received speaker honorary and research grants for other research projects from P&G Health Germany. C.S. is employed by BioTeSys GmbH, Esslingen. E.R. and J.G. have no conflict of interests to declare.

## Author Contributions

R.O. measured serum folate forms, conducted data analysis, and drafted the paper. E.R. participated in data acquisition and provided critical input to the manuscript. C.S. planned and conducted the original study and provided critical input to the content. J.G. supervised the study and provided critical input to the content.

## Supporting information



Supporting information

## Data Availability

Data can be provided in aggregated form to scientists interested in the topic upon request submitted to the corresponding author.
