# Supplementary material for: Serum Concentrations of Folate Forms Following Supplementation of Multimicronutrients with 400 µg or 800 µg Mix of (6S)‐5‐Methyltetrahydrofolate and Folic Acid (1:1) in Women of Childbearing Age
Source: Mol Nutr Food Res. 2024 Oct 28;68(22):2400444. doi: 10.1002/mnfr.202400444 (PMC11605785; doi:10.1002/mnfr.202400444)
Supplement: Supplementary file 1 — Supporting information [file MNFR-68-2400444-s001.docx]

**Serum concentrations of folate forms following supplementation of multimicronutrients with 400 µg or 800 µg mix of (6S)-5-methyltetrahydrofolate and folic acid (1:1) in women of childbearing age**

**Supplemental Tables**

| **Supplemental Table 1.** Nutrient’s composition of one portion (daily dose) of the study products. | | | |
| --- | --- | --- | --- |
| **Ingredients** | **Elevit® Gynvital capsule^a^** | **Femibion® 1 tablet^b^** | **Difference (Femibion® vs. Elevit® Gynvital)** |
| Folate | 400 µg Folic acid/ Metafolin | 800 µg Folic acid/ Metafolin | + 400 µg folate |
| Vitamin B1 | 1.4 mg | 1.2 mg | - 0.2 mg vitamin B1 |
| Vitamin B2 | 1.4 mg | 1.6 mg | +0.2 mg vitamin B2 |
| Vitamin B6 | 1.9 mg | 1.9 mg | = |
| Vitamin B12 | 2.6 µg | 3.5 µg | + 0.9 µg vitamin B12 |
| Biotin | 30 µg | 60 µg | + 30 µg biotin |
| Niacin | 18 mg | 15 mg | - 3 mg niacin |
| Pantothenic acid | 6 mg | 6 mg | = |
| Vitamin C | 85 mg | 110 mg | +25 mg vitamin C |
| Vitamin E | 10 mg | 13 mg | + 3 mg vitamin E |
| Vitamin A | 770 µg | - | -770 µg vitamin A |
| Vitamin D | 5 µg / 200 IU | 20 µg / 800 IU | + 15 µg vitamin D |
| Iodine | 150 µg | 150 µg | = |
| Copper | 1000 µg | - | -1000 µg |
| Iron | 14 mg | - | -14 mg |
| Magnesium | 57 mg | - | -57 mg |
| Selenium | 60 µg | - | -60 µg |
| Zinc | 10 mg | - | -10 mg |
| Omega-3-fatty acid | 200 mg | - | -200 mg |
| ^a^ Lot Number: MA029U8, Storage life: 03/2017.  ^b^ Lot Number: 488615/090, Storage life: 11/2017. | | | |

| **Supplemental Table 2.** Main characteristics of the 172 participating women. | | | | |  |
| --- | --- | --- | --- | --- | --- |
|  | All | 400 µg/day mix of (6S)-5-CH3-H4folate-Ca and folic acid (1:1) | 800 µg/day mix of (6S)-5-CH3-H4folate-Ca and folic acid (1:1) | P | |
| Number | 172 | 83 | 89 | - | |
| Age, years | 26.9 (6.5) | 26.7 (5.6) | 27.1 (7.2) | 0.630 | |
| BMI, kg/m² | 22.0 (2.7) | 21.9 (2.8) | 22.1 (2.7) | 0.575 | |
| Hb, g/dl | 13.2 (0.9) | 13.3 (0.9) | 13.0 (0.9) | 0.100 | |
| Hct, % | 39.4 (2.6) | 39.8 (2.5) | 39.1 (2.6) | 0.060 | |
| MCV, fl | 87.6 (4.5) | 87.8 (4.3) | 87.4 (4.7) | 0.570 | |
| GOT, U/l | 18.5 (6.2) | 18.5 (6.5) | 18.5 (6.0) | 0.981 | |
| Creatinine, mg/dl | 0.8 (0.1) | 0.8 (0.1) | 0.8 (0.1) | 0.665 | |
| Data are shown as mean (SD).  GOT, Glutamat-Oxalacetat-Transaminase; MCV, mean corpuscular volume; BMI, body mass index; Hct, hematocrit; Hb, hemoglobin. | | | | |  |

.
